# Daidzein and Genistein: Natural Phytoestrogens with Potential Applications in Hormone Replacement Therapy

**DOI:** 10.3390/ijms26146973

**Published:** 2025-07-20

**Authors:** Aekkhaluck Intharuksa, Warunya Arunotayanun, Mingkwan Na Takuathung, Siripat Chaichit, Anchalee Prasansuklab, Kamonwan Chaikhong, Buntitabhon Sirichanchuen, Suthunya Chupradit, Nut Koonrungsesomboon

**Affiliations:** 1 Department of Pharmaceutical Sciences, Faculty of Pharmacy, Chiang Mai University, Chiang Mai 50200, Thailand; aekkhaluck.int@cmu.ac.th (A.I.); siripat.chaichit@cmu.ac.th (S.C.); 2 Kanchanabhishek Institute of Medical and Public Health Technology, Faculty of Public Health and Allied Health Science, Praboromarajchanok Institute, Nonthaburi 11150, Thailand; 3 Department of Pharmacology, Faculty of Medicine, Chiang Mai University, Chiang Mai 50200, Thailand; mingkwan.n@cmu.ac.th (M.N.T.); nut.koonrung@cmu.ac.th (N.K.); 4 Clinical Research Center for Food and Herbal Product Trials and Development (CR-FAH), Faculty of Medicine, Chiang Mai University, Chiang Mai 50200, Thailand; 5 College of Public Health Sciences, Chulalongkorn University, Bangkok 10330, Thailand; anchalee.pr@chula.ac.th; 6 Center of Excellence on Natural Products for Neuroprotection and Anti-Ageing, Chulalongkorn University, Bangkok 10330, Thailand; 7 Interdisciplinary Graduate Program in Biomedical Science, Graduate School, Chulalongkorn University, Bangkok 10330, Thailand; kamonwan.chaikhong@gmail.com; 8 Department of Pharmaceutical Care, Faculty of Pharmacy, Chiang Mai University, Chiang Mai 50200, Thailand; buntitabhon.s@cmu.ac.th (B.S.); suthunya.chu@cmu.ac.th (S.C.); 9 Center for Medical and Health Technology Assessment (CM-HTA), Department of Pharmaceutical Care, Faculty of Pharmacy, Chiang Mai University, Chiang Mai 50200, Thailand

**Keywords:** estrogen, flavonoids, *Glycine max*, gynecology, isoflavone, menopause, phytochemical, soybean

## Abstract

Menopause is characterized by a decline in estrogen levels, leading to symptoms such as vasomotor instability, osteoporosis, and increased cardiovascular and cognitive risk. Hormone replacement therapy (HRT) remains the gold standard for managing menopausal symptoms; however, concerns regarding its long-term safety, including elevated risks of cancer and cardiovascular events, have prompted interest in alternative therapies. Phytoestrogens, particularly the isoflavones daidzein and genistein, are plant-derived compounds structurally similar to 17β-estradiol (E2) and capable of binding estrogen receptors. Found abundantly in soybeans and red clover, these compounds exhibit selective estrogen receptor modulator (SERM)-like activity, favoring ERβ over ERα, which underlies their tissue-specific effects. In vitro, in silico, and in vivo studies demonstrate their ability to modulate estrogenic pathways, inhibit oxidative stress, and influence reproductive and neurological function. Clinical trials show that daidzein and genistein, especially in equol-producing individuals, can reduce vasomotor symptoms such as hot flashes and night sweats. While results across studies vary, consistent findings support their safety and modest efficacy, particularly for women unable or unwilling to use HRT. Pharmacokinetic studies reveal moderate bioavailability and interindividual variability due to gut microbiota metabolism. At dietary levels, these compounds are generally safe, although high-dose supplementation is discouraged in individuals with hormone-sensitive cancers. Emerging evidence suggests lifelong consumption of soy-based foods may reduce cancer risk. In conclusion, daidzein and genistein represent promising, well-tolerated natural alternatives to conventional HRT, offering symptom relief and additional health benefits. Further research is warranted to optimize dosing, improve clinical outcomes, and clarify long-term safety in diverse populations, particularly with genetic variations in isoflavone metabolism.

## 1. Introduction

Menopause is marked by a decline in estrogen levels and the permanent cessation of menstrual cycles for at least 12 consecutive months, which leads to symptoms such as vasomotor instability, osteoporosis, cardiovascular risk, and cognitive changes [1]. Current guidelines on hormone replacement therapy (HRT) provide healthcare professionals with evidence-based recommendations for managing menopausal symptoms, emphasizing individualized care based on patient-specific factors [2,3,4,5,6,7,8]. HRT, comprising estrogen alone or in combination with progestin, is the most prescribed therapy. It is available in various forms, including tablets, gels, patches, and implants. Evidence supports HRT’s benefits in alleviating menopausal symptoms, preventing osteoporosis, and may reduce the risk of some conditions such as type 2 diabetes [9] and dementia [10]. However, the cancer and cardiovascular risks associated with HRT vary depending on the type and time of administration [7]. Key principles of HRT use include short-term treatment (typically less than five years) for symptomatic relief in women without contraindications, the lowest effective dose to minimize adverse effects, and the consideration of non-hormonal or herbal alternatives for women with contraindications.

### 1.1. Hormone Replacement Therapy (HRT) for Menopausal Symptoms and Related Symptoms

The American College of Obstetricians and Gynecologists recommends HRT for symptomatic relief of menopausal symptoms, including hot flashes and vaginal atrophy, and supports its use beyond age 65 in appropriate cases [2]. In contrast, the North American Menopause Society suggests that, for women under 60, the benefits of HRT generally outweigh the risks [6]. Similarly, the Endocrine Society states that, when initiated during perimenopause or the early years of menopause, HRT presents lower risks than previously believed and may reduce all-cause mortality [3]. However, the U.S. Preventive Services Task Force advises against the use of estrogen alone or combined estrogen–progestin therapy for the primary prevention of chronic conditions, such as coronary heart disease, in postmenopausal women, citing evidence that the potential risks outweigh the benefits [7]. The National Institute for Health and Care Excellence has recently updated its menopause guidelines, recommending HRT as the first-line treatment for menopausal symptoms, including hot flushes, night sweats, depression, and sleep disturbances [8]. This update recommends menopause-specific cognitive behavioral therapy as an option for individuals aged 40 years and older in addition to HRT, or as an alternative for those who prefer not to take HRT or for whom it is contraindicated.

Beyond symptom management, HRT also plays a role in bone health, particularly in preventing osteoporosis and reducing fracture risk. The UK National Osteoporosis Guideline Group states that HRT can help strengthen bones and lower the likelihood of fractures in individuals with osteoporosis or a high risk of fractures [5]. The Endocrine Society’s clinical practice guideline on osteoporosis management in postmenopausal women recommends considering HRT for women under 60 or within 10 years of menopause onset, particularly those at high fracture risk with additional climacteric symptoms [4]. Estrogen-only therapy is advised for women without a uterus, while combined estrogen–progestogen therapy is recommended for those with an intact uterus. However, HRT is contraindicated in individuals with a history of breast cancer, cardiovascular disease, or thromboembolic disorders. Nonetheless, HRT currently remains the primary treatment for alleviating menopausal symptoms, maintaining bone density, and reducing fracture risk, with treatment decisions tailored to individual risk factors, symptom severity, and patient preferences [11]. Regular re-evaluation is essential to ensure the benefits of HRT continue to outweigh its risks. However, concerns regarding HRT’s long-term safety have fueled interest in alternative therapies, including complementary botanicals and natural products such as phytoestrogens, herbal remedies, or vitamins.

### 1.2. Natural Product and Phytoestrogen: The Alternative Hormone Replacement Therapy

Natural products have long served as alternative therapies and sources of lead compounds for treating various diseases. This has encouraged ongoing research into plant-based treatments for challenging conditions such as cancer and age-related disorders, where conventional treatments may be insufficient or carry significant risks [12,13,14]. In recent years, there has been an increasing trend toward the use of natural sources for HRT, like herbal supplements and plant products, largely driven by heightened awareness of the risks and limitations associated with conventional hormone regimens [15,16]. Commonly prescribed synthetic hormones, such as conjugated equine estrogens and medroxyprogesterone acetate, have been associated with various adverse effects, including an elevated risk of breast cancer [17]. In addition to safety concerns, ethical objections regarding the treatment of horses used in the production of conjugated equine estrogens and the structural dissimilarity between these synthetic hormones and their human endogenous counterparts have further intensified public and scientific interest in safer alternatives [17]. Consequently, a substantial proportion of women—estimated at from 40% to 50% in Western countries—now seek complementary approaches to healthcare, with many adopting plant-based therapies as part of their treatment strategies [18,19]. This shift has led to increased research efforts aimed at discovering and developing natural compounds for use in HRT. Plant-derived alternatives are being actively investigated for their potential to offer safer, more biocompatible options for hormonal support, particularly in managing menopause-related symptoms [18].

Among natural compounds, phytoestrogens have received considerable attention. These are plant-derived molecules capable of interacting with the mammalian endocrine system [20,21,22]. Phytoestrogens are non-steroidal polyphenolic secondary metabolites that exert a range of biological effects, primarily due to their structural similarity to 17β-estradiol, the primary estrogen in humans [23]. This resemblance allows them to bind to estrogen receptors and either mimic or modulate the physiological actions of endogenous estrogens [24,25]. In vivo, they may interfere with hormone-regulated pathways by competing with endogenous estrogens for receptor binding sites [26]. Beyond their interaction with estrogen receptors, phytoestrogens may also exert biological effects through non-estrogen receptor-mediated mechanisms, including modulation of cellular signaling pathways, gene expression, and enzymatic activities [27,28]. This dual mode of action contributes to their therapeutic potential in managing hormone-related conditions such as menopausal symptoms, osteoporosis, and hormone-dependent cancers [29,30]. The safety of phytoestrogens remains a subject of concern. To the best of our knowledge, randomized controlled trials investigating the safety of phytoestrogens are still limited. However, recent data from the Netherlands national database and the World Health Organization (WHO) adverse reaction (AR) spontaneous reporting system indicate a range of mild to serious ARs associated with phytoestrogen-containing products. The most frequently reported ARs were mild, including nausea, pruritus, and pyrexia. Nonetheless, there have also been reports of postmenopausal bleeding, which may be linked to the long-term use of products with phytoestrogenic activity [31].

Isoflavones, particularly daidzein and genistein, are among the most studied phytoestrogens due to their abundance in the human diet and their well-documented biological activity. These compounds are predominantly found in a variety of fruits, vegetables, and whole grains [32,33]. Other important classes of phytoestrogens include lignans [33], coumestans [34], chromenes [35,36,37], prenylnaringenins [38,39,40], and stilbenes [41]. Among these, genistein and daidzein are considered potent phytoestrogens, as their structural similarity to 17β-estradiol (E2) enables high-affinity binding to estrogen receptors, particularly ERβ, resulting in pronounced estrogen-like effects and diverse biological activities [24,42,43]. Given their dietary prevalence and potent biological effects, daidzein and genistein are the primary focus of the present study, which aims to explore their potential roles in modulating hormone-related physiological processes.

## 2. Results

### 2.1. Daidzein and Genistein: Chemical Structure, Chemical Information

**Figure 1:**
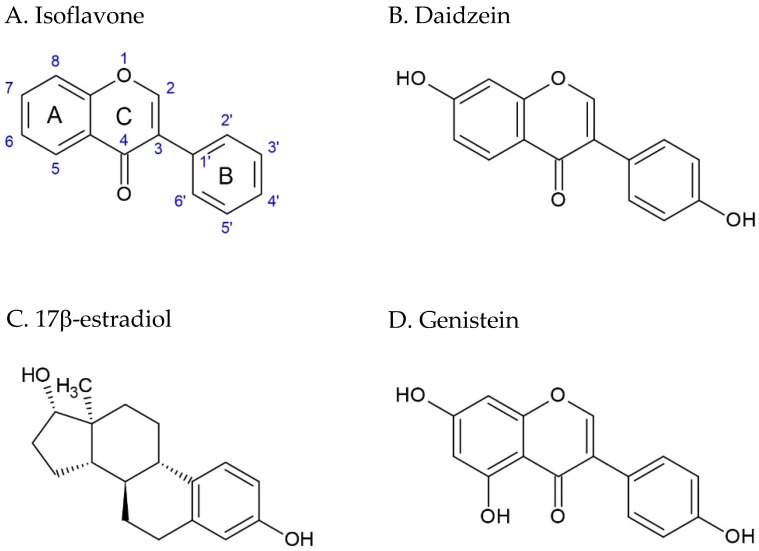
Molecular structures of (**A**) isoflavone, (**B**) daidzein, (**C**) 17β-estradiol, and (**D**) genistein, highlighting shared aromatic rings and 4′-hydroxyl group, with genistein differing by the presence of a 5-hydroxyl group on ring A.

Daidzein (IUPAC name: 7-hydroxy-3-(4-hydroxyphenyl)chromen-4-one) (Figure 1B) and genistein (IUPAC name: 5,7-dihydroxy-3-(4-hydroxyphenyl)chromen-4-one) (Figure 1D) are naturally occurring isoflavones (Figure 1A), a subclass of flavonoids characterized by a C6-C3-C6 carbon framework [44]. Structurally, isoflavones are composed of two aromatic rings (designated as rings A and B) and a heterocyclic ring (ring C), as illustrated in Figure 1. These compounds are commonly found in plants in their glycosylated forms, known as glycones, where a sugar moiety is covalently linked to the hydroxyl group at the 7-position via a glycosidic bond. Representative examples include genistein and daidzein, the glycosidic counterparts of genistein and daidzein, respectively. Upon enzymatic or chemical hydrolysis, these glycosides are converted into their corresponding aglycones—genistein and daidzein—which are the bioactive forms responsible for various physiological effects [45,46].

A key structural distinction between genistein and daidzein lies in the presence of an additional hydroxyl group at the 5-position of ring A in genistein, which may influence its biological properties. The structure–activity relationships (SARs) of genistein and daidzein reveal that the hydroxyl group at the 4′-position and the 7-position groups partially mimic the functional groups of E2 (Figure 1C), enabling the hydrogen bonding necessary for estrogen receptor (ER) binding. While both compounds exhibit this molecular mimicry, variations in hydroxylation patterns contribute to differential receptor binding affinities and selectivity, influencing their biological outcomes [47,48,49,50]. Both genistein and daidzein show preferential binding to ERβ over ERα, largely due to the tighter fit within the smaller and more compact ligand-binding domain of ERβ. The 5-hydroxyl group in genistein contributes to intramolecular stabilization and promotes an optimal fit within the ERβ binding pocket, thereby conferring greater ERβ selectivity relative to daidzein [51,52]. Due to their ability to modulate estrogenic pathways, genistein and daidzein are classified as phytoestrogens and have attracted significant attention for their potential therapeutic roles in managing hormone-related disorders, including menopausal symptoms, osteoporosis, and estrogen-dependent malignancies. Additionally, these aglycones may undergo further metabolic transformation within the body, leading to the formation of biologically active metabolites such as equol, a derivative of daidzein, which may exert enhanced or distinct effects through interaction with estrogen receptors and other molecular targets [53].

### 2.2. Source of Daidzein and Genistein

Isoflavones, particularly daidzein and genistein, are among the most extensively studied phytoestrogens and are predominantly found in soybeans (*Glycine max* L.), which serve as a major dietary source of these compounds [54,55]. In addition to daidzein and genistein, soybeans also contain glycitein. These isoflavones exist in both aglycone forms (daidzein, genistein, and glycitein) and glycoside forms (daidzin, genistin, and glycitin), with the glycosylated derivatives typically hydrolyzed in the gastrointestinal tract to release bioactive aglycones that exert estrogenic and other physiological effects [54]. However, isoflavones are not synthesized by plants solely for human health benefits. In plants, they play critical ecological roles, functioning as phytoalexins that help deter pathogenic fungal infections [56,57]. Furthermore, they act as key signaling molecules in the establishment of symbiotic relationships between leguminous plants and nitrogen-fixing bacteria in root nodules, thereby facilitating biological nitrogen fixation [58,59,60].

The concentrations of daidzein and genistein in soybeans can vary significantly depending on cultivar, geographic origin, and environmental conditions. On average, raw mature soybean seeds contain approximately 0.6 mg/g of daidzein and 0.8 mg/g of genistein [61]. Yue et al. further reported that these isoflavones are distributed across different anatomical parts of the soybean seed, including the cotyledon, seed coat, and germ [62]. Beyond whole soybeans, a variety of soy-based food products also contain high levels of these compounds. Non-fermented products such as soy flour [32,63], tofu [64], soy milk [54,64], as well as fermented soy products, namely, miso [32,65], natto [66], and tempeh [32,67], are recognized as rich dietary sources of isoflavones. In addition to soybeans, red clover (*Trifolium pratense* L.) is another important plant source of daidzein and genistein [68,69,70]. It also contains significant amounts of other isoflavones such as formononetin and biochanin A. Saviranta et al. investigated the distribution of isoflavones in different plant tissues of red clover, including flower buds, young flowers, leaves, stems, and roots, and found the highest concentrations of daidzein and genistein in the petioles, ranging from 0.11 to 0.28 mg/g and from 0.30 to 0.54 mg/g, respectively [68]. These isoflavones have also been detected in other *Trifolium* species, including *T. repens* L., *T. medium* L., *T. rubens* L., and *T. pannonicum* Jacq. [71]. Several additional studies have reported the presence of daidzein and genistein in a range of commonly consumed vegetables [72], fruits [73], seeds [73,74], and cereals [75,76], indicating their broader dietary relevance. Moreover, these compounds have been identified in other plant species, including pistachio nuts (*Pistacia vera* L. var. *bronte*) [77], kudzu (*Pueraria montana* (Lour.) Merr.) [78,79], white Kwao Krua (*Pueraria candollei* Wall. ex Benth. and *P. mirifica* Airy Shaw & Suvat.) [35,36,80,81], and red Kwao Krua (*Butea superba* Roxb. ex Willd.) [82].

### 2.3. Bioactivities: Estrogenic Activity of Daidzein and Genistein

#### 2.3.1. In Silico

Estrogens are sex hormones that play a pivotal role in the development of secondary sex characteristics. Beyond their reproductive roles, estrogens significantly influence various physiological functions in multiple organ systems, including the cardiovascular system, liver, pancreas, bone, brain, and immune system [83]. These effects are primarily mediated through estrogen receptors (ERs) [84]. ERs are nuclear receptors classified into two main subtypes, estrogen receptor alpha (ERα) and estrogen receptor beta (ERβ). ERs are distributed across multiple tissues, including reproductive organs, breast tissue, and various cancer cells. While some tissues express both ERα and ERβ, others exhibit only one subtype [85]. Given that estrogenic activity relies on ER-mediated signaling pathways, alterations in the expression levels of ERα and ERβ can be associated with various disease states.

ERs exhibit a conserved structural framework that supports their similar biological functions. However, ERα and ERβ differ in several key aspects, including specific amino acid residues, domain organization, and ligand-binding affinities. Both receptor subtypes consist of five principal domains contributing to their function [86]. The N-terminal A/B domain contains the activation function-1 (AF-1), regulating ligand-independent transcriptional activity. The highly conserved C domain, or DNA-binding domain (DBD), facilitates receptor binding to estrogen response elements (EREs) within DNA. The D domain serves as a flexible hinge linking the DBD to the ligand-binding domain. The E domain, also known as the ligand-binding domain (LBD), is responsible for ligand-dependent transcriptional activation and mediates interactions with co-regulatory proteins. The less conserved terminal F domain modulates receptor stability and protein–protein interactions [86,87]. These structural differences between ERα and ERβ contribute to their distinct tissue distributions and functional roles in physiological and pathological processes. While ERα and ERβ possess a comparable domain architecture and are both capable of binding estrogen and activating transcription through estrogen response elements (EREs), ERβ exhibits a weaker activation function in its AF-1 domain compared to ERα [88]. However, the AF-2 domain functions similarly in both receptor subtypes. In addition, the ligand-binding pocket of ERβ is approximately 20% smaller than that of ERα, which may contribute to differences in ligand selectivity and pharmacological behavior [89,90].

**Figure 2:**
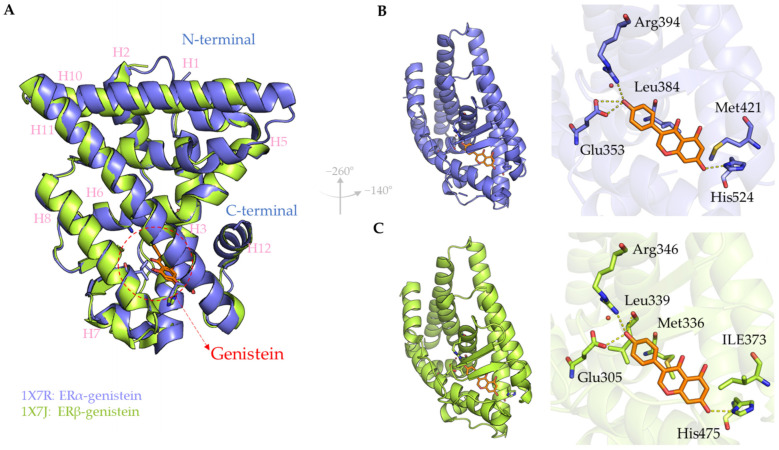
Binding interactions of genistein with estrogen receptor alpha (ERα) and estrogen receptor beta (ERβ). (**A**) Superimposed structures of genistein (orange) with ERα (blue ribbon) and ERβ (green ribbon). (**B**) Binding pose of genistein within the ERα LBD and within (**C**) ERβ LBD, highlighting key amino acid interactions. Hydrogen bonds are depicted as yellow dashed lines. The graphical representation was generated using PyMOL2.

Genistein exhibits a binding orientation to ERs that closely resembles that of E2, particularly within the ERβ LBD [90] (Figure 2A). Its phenolic ring mimics the A ring of E2, forming critical hydrogen bonds with Glu305 and Arg346, while the 7-OH group of genistein engages with His475, contributing to stable interactions within the binding cavity (Figure 2C). For the binding to ERα, the 4′-OH group of genistein forms hydrogen bonds with Leu339, Glu353, and Arg394, while the 7-OH group interacts with His524 (Figure 2B). Although the binding modes in ERα and ERβ appear nearly identical, subtle structural and electronic differences—such as the replacement of ERα Leu384 with ERβ Met336 and ERα Met421 with ERβ Ile373—modulate ligand selectivity. The smaller cavity size of ERβ, coupled with the planar structure of genistein, allows for tighter packing and more favorable van der Waals interactions [89,90]. Ab initio quantum chemical calculations further support that genistein preferentially interacts with bulkier Met336 in ERβ over Leu384 in ERα due to more favorable positioning and electronic compatibility [51]. While no significant repulsion was observed in the crystal structure between the 5-OH of genistein and the Met421 of ERα, the potential for differential interaction exists [51]. These findings suggest that modifying the genistein scaffold to exploit these residue-specific interactions could further enhance ERβ selectivity.

Although a co-crystallized structure of daidzein bound to human estrogen receptors (ERs) has yet to be reported, recent in silico studies have provided significant insights into its binding behavior with ERα and ERβ. These computational investigations underscore daidzein’s potential as a selective estrogen receptor modulator (SERM), highlighting both its affinity and selectivity toward ER subtypes. Satpathy and colleagues reported that daidzein forms hydrogen bonds with key amino acid residues in both ERα and ERβ, with interaction profiles resembling those observed for genistein [91]. In addition to hydrogen bonding, hydrophobic interactions near the benzene rings of daidzein were found to contribute to the stabilization of the ligand within the receptor binding pocket. Notably, docking simulations yielded a slightly higher binding affinity for ERβ, as reflected by more favorable docking scores [91]. Complementary computational analyses further suggest that daidzein may exhibit stronger binding affinity to ERα than E2, primarily due to its hydroxyl substitutions and planar aromatic scaffold, which facilitate favorable interactions within the hydrophobic cleft of the receptor [92]. Molecular modeling of daidzein and its metabolites has also revealed that the positioning of hydroxyl groups plays a critical role in modulating its binding energy and affinity, particularly toward ERβ [93]. Interestingly, these structural features may account for the compound’s receptor subtype selectivity. However, experimental studies indicate that daidzein exerts a greater inhibitory effect on ERα compared to ERβ, resulting in a decreased ERα/ERβ expression ratio in treated cells [94].

Together, these computational studies support the therapeutic relevance of daidzein and genistein in modulating estrogen receptor-mediated pathways. Their binding characteristics reinforce their application in the management of hormone-related conditions such as menopause, osteoporosis, and hormone-dependent cancers. Moreover, the insights gained from these studies provide a valuable foundation for the rational design and optimization of phytoestrogen-derived compounds with enhanced receptor selectivity and pharmacological efficacy.

#### 2.3.2. In Vitro

Genistein and daidzein have attracted significant attention due to their ability to bind ERs and modulate estrogenic signaling pathways. Their structural similarity to E2 allows these compounds to function as phytoestrogens with SERM-like properties. Genistein and daidzein exhibit tissue-specific agonist or antagonist activities depending on the receptor subtype expression and cellular context [95,96]. In vitro studies have played a pivotal role in characterizing their estrogenic potential, particularly in hormone-responsive systems.

A key mechanism underlying the estrogenic effects of these phytoestrogens involves their interaction with the classical nuclear ERs, ERα and ERβ [97]. Competitive binding assays have consistently demonstrated that genistein exhibits more than a 20-fold greater affinity for ERβ than for ERα, with daidzein also showing a preferential binding toward ERβ [52,98,99]. Further support for ERβ selectivity comes from fluorescence resonance energy transfer (FRET) assays, which have demonstrated that genistein, daidzein, and related phytoestrogens more efficiently recruit coactivators such as steroid receptor coactivator 3 (SRC3) to ERβ than to ERα. This reinforces their preference for ERβ at the level of interacting with coregulator proteins [100].

Functional studies using estrogen-responsive cells have confirmed the estrogenic activity of both genistein and daidzein. In MCF-7 breast cancer cells, which express the ERα, low concentrations of genistein and daidzein stimulate cell proliferation, similar to the growth-promoting effects of endogenous estrogens. In contrast, at higher concentrations, these compounds inhibit proliferation, indicating a biphasic, dose-dependent effect. These findings suggest that ERα is critical for mediating the growth-promoting actions of these isoflavones, whereas ERβ may antagonize ERα-mediated proliferation and contribute to tumor suppression [100,101].

The role of genistein and daidzein in regulating estrogen metabolism has also been explored. Both compounds have demonstrated the ability to inhibit enzymes involved in steroid hormone processing, such as cytochrome P450 3A4 (CYP3A4), 17β-hydroxysteroid dehydrogenase (17β-HSD), sulfotransferases (SULTs), and UDP-glucuronosyltransferases (UGTs). Inhibiting these metabolic pathways may lead to increased intracellular levels of active estrogens or their precursors, thereby enhancing ER-mediated signaling [102,103,104,105].

In vitro assays using Ishikawa cells further support the estrogenic potential of genistein and daidzein in reproductive tissues. Both isoflavones increase alkaline phosphatase activity, an established marker of estrogen receptor activation, with genistein displaying greater potency. These observations reinforce their capacity to mimic estrogenic effects in hormone-sensitive endometrial tissue [106,107].

Reporter gene assays, including mammalian cell systems and yeast estrogen screens (YES assays), have been instrumental in confirming the estrogenic activity of genistein and daidzein. These assays typically involve the expression of ERs linked to a reporter gene, with activation by isoflavones resulting in quantifiable reporter expression [107,108]. Collectively, these models confirm that both genistein and daidzein can activate estrogen receptors and elicit downstream responses, although their potencies are significantly lower than that of estradiol.

In addition to classical ER pathways, genistein and daidzein are capable of activating the G-protein-coupled estrogen receptor (GPER), also known as GPR30, which mediates rapid, non-genomic estrogen signaling [84,109,110]. Genistein has been identified as a GPER agonist, exhibiting an IC_50_ of approximately 133 nM [111]. Activation of GPER by genistein initiates a cascade of intracellular second messenger events [112]. These processes subsequently lead to transactivation of the epidermal growth factor receptor (EGFR), triggering downstream signaling via the PI3K/Akt and MAPK/ERK signaling pathways, contributing to estrogenic effects independent of direct gene transcription [113,114]. Daidzein also exhibits notable estrogenic activity, including the activation of GPER-mediated physiological pathways. Kajta et al. demonstrated that daidzein-induced activation of GPER signaling attenuated glutamate-induced neurotoxicity and pro-apoptotic signaling in neuronal cells, highlighting its potential role in modulating estrogen-responsive neuroprotective pathways [115].

**Figure 3:**
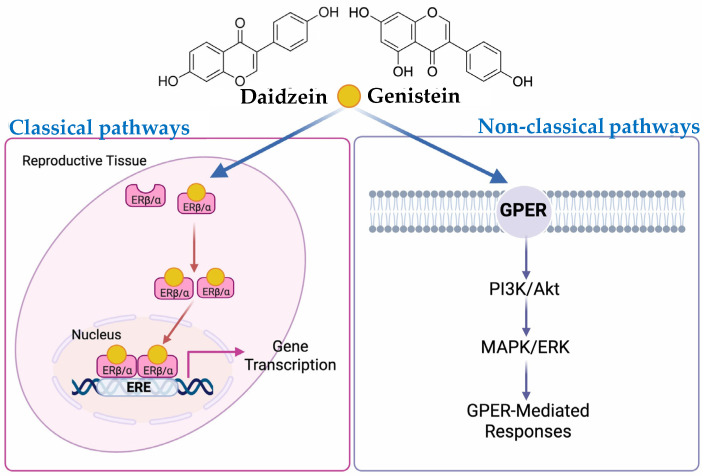
Classical and non-classical pathways of daidzein and genistein signaling. Created using BioRender by Aekkhaluck Intharuksa. Available at https://BioRender.com/3xgx6l4 (accessed on 29 June 2025).

Taken together, the in vitro evidence underscores the role of genistein and daidzein as functional phytoestrogens capable of modulating estrogen receptor activity through both classical and non-classical pathways, as illustrated in Figure 3. Their selective affinity for ERβ, ability to influence ERα-mediated cell proliferation, and engagement of GPER-related signaling suggest their potential utility in hormone-related therapeutic applications, particularly where selective estrogenic modulation is desired.

#### 2.3.3. In Vivo

Emerging preclinical evidence highlights the complex duality of genistein and daidzein in HRT, where their biological effects depend critically on dosage and tissue specificity. In ovariectomized rat models, low-dose genistein (typically 10 mg/kg/day orally) has been shown to improve ovarian morphology and reduce oxidative stress through cAMP-PKA signaling pathways that restore glutathione levels in granulosa cells [116]. However, higher doses of genistein (20–100 mg/kg/day or above) can disrupt endocrine balance, suppress luteinizing hormone-stimulated progesterone production, and hyperactivate the hypothalamic–pituitary–gonadal axis via enhanced GnRH secretion, influenced by changes in kisspeptin receptors and epigenetic regulators such as SIRT1 and MKRN3 [117]. Translating genistein doses from animal studies to human equivalents involves normalization based on body surface area, which accounts for differences in metabolism and physiology between species. For instance, a commonly used low dose in rat studies, 10 mg/kg/day, translates to approximately 1.62 mg/kg/day in humans, equating to about 113 mg/day for a 70 kg adult when using standard conversion factors. Higher experimental doses in rats, ranging from 20 to 100 mg/kg/day, correspond to human equivalent doses of from 3.24 to 16.2 mg/kg/day, or from roughly 227 to 1134 mg/day for a 70 kg adult [118]. In real-world dietary scenarios, human intake of genistein is substantially lower than these calculated equivalents. Individuals consuming high-soy diets may ingest up to 50–100 mg of genistein per day, while those following typical Western diets generally consume less than 10 mg per day [119]. It is also important to recognize that the plasma concentrations of genistein achieved in rodents at these experimental doses are often significantly higher than those observed in humans consuming genistein through diet alone. Therefore, direct extrapolation of animal data to human health outcomes should be approached with caution, as differences in absorption, metabolism, and exposure levels can influence the biological effects observed. This dose-dependent dichotomy emphasizes the precision required in phytoestrogen administration to balance therapeutic benefits against adverse effects [120,121].

The osteoprotective potential of these compounds aligns with classical HRT mechanisms, where estrogen suppresses osteoclast activity to preserve bone density. Daidzein emerges as particularly promising, outperforming genistein in preventing trabecular bone loss in ovariectomized rats with efficacy comparable to synthetic estrogens like 17α-ethinylestradiol [122]. This enhanced efficacy may be attributed to daidzein’s ability to modulate several key molecular pathways involved in bone remodeling more effectively. Most notably, daidzein significantly increases osteoprotegerin (OPG) expression while suppressing receptor activator of nuclear factor kappa-B ligand (RANKL), resulting in a lower RANKL/OPG ratio that inhibits osteoclast differentiation and bone resorption, a mechanism where genistein shows only modest effects [123]. In addition, daidzein activates both estrogen receptor subtypes (ERα and ERβ) in osteoblasts, enhancing osteogenic gene expression and further suppressing pro-osteoclastic cytokines, such as interleukin-6, whereas genistein predominantly acts via ERβ, limiting its anabolic impact [124]. Recent studies also reveal that daidzein, unlike genistein, promotes angiogenesis–osteogenesis coupling through the Caveolin-1/EGFR/AKT pathway, supporting H-type vessel formation critical for bone health [125]. These combined actions explain why daidzein more effectively preserves trabecular bone microarchitecture and prevents bone loss compared to genistein. These preclinical findings align with clinical observations that early postmenopausal HRT reduces fracture risk by maintaining bone mineral density, positioning daidzein as a viable alternative for osteoporosis prevention without the uterotrophic risks associated with traditional estrogen therapy.

Beyond skeletal benefits, genistein and daidzein demonstrate protective roles in urogenital health. Estrogen deficiency exacerbates oxidative damage in bladder tissue following ischemia/reperfusion injury, but both phytoestrogens replicate estrogen’s ability to mitigate detrusor muscle dysfunction [126]. By reducing oxidative markers like malondialdehyde and modulating inflammatory pathways such as TGF-β, they restore bladder contractility—a critical consideration for postmenopausal women prone to urogenital atrophy [126]. Their antioxidant capacity extends systemically, counteracting elevated reactive oxygen species in hepatic and neuronal tissues through enhanced superoxide dismutase activity and reduced lipid peroxidation [127]. Genistein’s tissue-specific action is particularly notable, as it restores granulosa cell glutathione via cAMP-PKA signaling, mirroring endogenous estrogen’s protective mechanisms [127].

Reproductive tissue responses reveal further complexity. Low-dose genistein increases uterine weight through PI3K/AKT-mediated reductions in H3K27 methylation, predisposing to hormone-driven disorders, while daidzein’s uterotrophic effects only manifest at higher concentrations [128]. Age-dependent interactions amplify these nuances, with genistein upregulating ovarian genes like CXCL-12 and EGR-1 more robustly in aging rats, suggesting divergent impacts on reproductive aging [129]. These findings gain translational relevance from historical livestock models, where phytoestrogen-rich diets caused reversible infertility in ewes and cheetahs—phenomena paralleling genistein’s inhibition of steroidogenesis in bovine granulosa cells [130]. Such cross-species observations underscore the importance of therapeutic windows and species-specific responses in HRT applications [131].

While genistein and daidzein exhibit selective estrogen receptor modulator (SERM)-like properties, their clinical translation requires cautious optimization. Daidzein’s preferential bone preservation and genistein’s dual roles in oxidative stress mitigation and ovarian function modulation present compelling therapeutic avenues [132]. However, genistein’s capacity to disrupt endocrine regulation at supraphysiological doses—evident in its suppression of LH-stimulated progesterone and stimulation of premature luteolysis in cattle—demands rigorous dose-response characterization [133,134]. Future research must bridge these preclinical insights to human trials, particularly given mixed clinical results on phytoestrogens’ efficacy in alleviating climacteric symptoms compared to classical HRT. In synthesizing these findings, genistein and daidzein emerge as multifaceted agents in postmenopausal health, balancing osteoprotection, urogenital support, and antioxidant activity against tissue-specific risks. Advancing their clinical utility will require refined dosing protocols and deeper mechanistic insights into their SERM-like properties to leverage therapeutic benefits while minimizing unintended consequences across diverse populations.

#### 2.3.4. Clinical Trials

Recent clinical and epidemiological research has broadened our perspective on the use of genistein and daidzein as alternatives to conventional HRT. Early preclinical laboratory studies raised concerns that low concentrations of these soy isoflavones might stimulate the proliferation of estrogen receptor-positive breast cancer cells, particularly through ERα-mediated pathways [126]. However, more robust evidence from large-scale epidemiological studies, notably the prospective cohort study by She et al. involving 5042 breast cancer survivors in China, found that higher soy food intake (measured as isoflavone consumption) was significantly associated with reduced breast cancer recurrence and mortality. This study, which followed participants for a median of 3.9 years, reported a hazard ratio of 0.68 (95% CI, 0.54–0.87) for recurrence among women in the highest quartile of soy intake compared to the lowest, indicating a statistically significant protective effect [135]. This apparent protective effect is thought to arise from genistein’s preferential activation of estrogen receptor beta (ERβ), which can counterbalance the proliferative influence of ERα and may contribute to tumor suppression. Additional anticancer mechanisms have been reported for genistein, including the inhibition of angiogenesis, induction of apoptosis, and modulation of cell cycle regulators [136].

Regarding the management of menopausal symptoms, clinical trials assessing genistein and daidzein have produced mixed results. Most studies indicate that isoflavone supplementation leads to only modest reductions in vasomotor symptoms, such as hot flashes, with the effect size typically smaller than that seen with conventional HRT [30]. Nevertheless, RCTs involving genistein-enriched preparations have demonstrated more promising outcomes. For example, randomized controlled trials (RCTs) with sample sizes ranging from 90 to over 200 participants have shown that daily supplementation with genistein (30–54 mg) can significantly reduce the frequency and severity of hot flashes compared to placebo, with some studies reporting reductions of up to 50% in symptom frequency. Notably, these effects appear more pronounced in women who are equol producers—individuals capable of metabolizing daidzein into equol, a more potent estrogenic compound. Despite these findings, not all RCTs have confirmed statistically significant benefits, highlighting the variability in individual responses and the need for further large-scale, well-controlled studies to clarify the therapeutic potential of soy isoflavones in menopausal symptom management [30].

The bone-protective properties of isoflavones are more consistently supported by clinical evidence [137]. A recent double-blind, placebo-controlled RCT enrolled 100 postmenopausal women, randomly assigning them to receive either a soy extract nutraceutical (*n* = 50) or placebo (*n* = 50) for 12 weeks. The study found that the soy extract group experienced significant improvements in bone turnover markers compared to the placebo, indicating a positive effect on bone health. Additionally, the soy extract group showed improvements in facial skin wrinkles and a significant reduction in total cholesterol, although no major differences were observed in menopausal symptoms or quality of life between groups [137]. Further support comes from systematic reviews and meta-analyses of RCTs. This consistently report that both genistein and daidzein have demonstrated the ability to reduce bone resorption and help maintain bone mineral density in postmenopausal women, with the most pronounced effects seen in interventions lasting at least 12 months and using genistein doses of 50 mg per day or higher [138,139,140]. While these benefits do not fully match the efficacy of classical HRT, they offer a promising natural alternative for women at risk of osteoporosis who cannot or choose not to use traditional hormone therapy.

In terms of safety, current clinical and epidemiological data are reassuring. There is no consistent evidence that dietary intake of genistein or daidzein increases the risk of breast or endometrial cancer in healthy women [126]. In fact, populations with high soy consumption tend to have lower rates of these cancers, and genistein’s activation of ERβ may play a protective role. However, the safety of high-dose isoflavone supplements, especially for women with a history of hormone-dependent cancers, has not been fully established, and caution is warranted until more long-term data are available [126].

In summary, the clinical evidence supports the safety of dietary genistein and daidzein and highlights their potential benefits for bone health and, to a lesser extent, menopausal symptom relief. Importantly, recent studies indicate that these phytoestrogens do not increase—and may even reduce—the risk of hormone-dependent cancers when consumed as part of a typical diet. However, their effectiveness as direct replacements for conventional HRT remains limited, and further long-term, well-controlled studies are needed to optimize dosing and clarify their role in diverse populations.

### 2.4. Other Bioactivities of Daidzein and Genistein

In addition to their well-established estrogenic and antiestrogenic activities, daidzein and genistein exhibit a wide range of biological effects through the modulation of intracellular signaling pathways. Their therapeutic potential has been demonstrated in cancer, cardiovascular disease, neurodegenerative disorders, and osteoporosis—age-related conditions that are particularly relevant to postmenopausal women. Table 1 and Table 2 highlight key findings from recent studies within the past five years that support their pharmacological relevance. These effects are primarily attributed to their potent antioxidant and anti-inflammatory properties [141,142,143,144], with additional data provided in the Table S1.

### 2.5. Pharmacokinetic and Toxicity

Daidzein and genistein are natural isoflavones commonly found in soy-based foods like tofu, tempeh, soy milk, and soybeans [179]. Isoflavones belong to a group of plant compounds called phytoestrogens, which have a chemical structure similar to human estrogen [180]. Due to this similarity, isoflavones are able to bind directly to estrogen receptors in the body, either mimicking or modulating the effects of natural estrogen [181]. Among the many isoflavones in soy, daidzein and genistein have been studied the most for their pharmacological effects, especially in relation to hormone-related conditions and chronic diseases [182,183]. Soy consumption is particularly common in Asian countries, where traditional diets are rich in soy products [184]. After ingestion, daidzein and genistein are absorbed in the small intestine either in their aglycone (non-sugar) forms or after their glycoside forms are broken down by enzymes [185,186]. After absorption, daidzein and genistein enter the portal vein and are transported to the liver, where phase II metabolism takes place [185]. Enzymes called UDP-glucuronosyltransferases (UGTs) and sulfotransferases (SULTs) carry out glucuronidation and sulfation [185]. These enzymes help turn the compounds into forms that dissolve better in water [185]. The main compounds found in plasma and urine are daidzein-7-glucuronide, genistein-7-glucuronide, and their sulfate forms [186,187]. Only a small amount stays in the active aglycone form [186]. After oral administration, the highest levels in the plasma are usually seen from within 6 to 8 h [188]. Although absorption is efficient, the overall bioavailability of daidzein and genistein remains low (approximately 20–30%) due to extensive first-pass metabolism in the liver and rapid elimination from the body [185,189]. Another important factor influencing pharmacokinetics is the presence of specific gut microbiota [190]. In some individuals, gut bacteria can metabolize daidzein into equol, a metabolite with potentially stronger and more selective estrogen receptor activity, especially for Erβ [188,191]. However, only 30–50% of people mainly from Asian populations are equol producers [191]. This variation may help explain why clinical outcomes differ between people and populations [190]. In addition, the recycling of conjugated isoflavones between the liver and intestine (called enterohepatic recirculation) can make them stay in the body longer [192]. The half-life of daidzein is between 6 and 10 h, while genistein has a half-life of from about 6 to 12 h [188,193]. These values may be influenced by diet, microbiota composition, liver enzyme activity, and co-administered medications [185,190].

Daidzein and genistein are considered selective estrogen receptor modulators (SERMs). Both estrogen receptor alpha (ERα) and estrogen receptor beta (ERβ) are binding targets for daidzein and genistein, with a stronger preference observed for Erβ [194]. ERα is mostly found in the uterus, breast, and liver, while ERβ is mainly present in the bone, brain, blood vessels, and prostate [194,195]. Because isoflavones can bind differently to each receptor, their effects vary depending on the tissue [194,195]. In tissues where ERα is more prevalent, such as the breast and uterus, the activation of this receptor can promote cell proliferation, resulting in concerns about an elevated cancer risk [196]. By contrast, ERβ activation is linked to suppressing inflammation, encouraging cell death, and halting cell growth [195]. This difference in receptor preference helps explain why daidzein and genistein can act as both protective agents and potential risks depending on the situation [194]. For instance, in bone tissue, isoflavones may help maintain bone mineral density in postmenopausal women by acting like estrogen when natural hormone levels decrease [197]. In the cardiovascular system, genistein can help maintain healthy blood vessel function by boosting nitric oxide production and lowering oxidative stress [182,198]. However, in hormone-sensitive breast cancer cells, low doses of genistein may promote cell growth through ERα activation, while higher doses might have the opposite effect by inhibiting cell growth [196].

Studies in humans and preclinical models have shown that daidzein and genistein are usually safe when eaten as part of a regular diet [199,200]. High doses of genistein did not cause major toxic effects in these studies [199,200]. Genistein toxicity appears only at very high doses that are not typically consumed through diet [200]. However, taking large amounts of daidzein and genistein for a long time may still have some risks, especially if they are taken as supplements. These compounds can work like weak estrogen, so they might affect how the hormone system in the body works. This is called endocrine disruption [201]. Animal studies show that large amounts of these isoflavones can make the uterus heavier and affect other reproductive organs [202,203]. Although these findings are mainly based on animal data and are not commonly seen with dietary intake in humans, there are still concerns about hormone-related effects in people who are more sensitive. Also, taking high doses may affect thyroid function, especially in people who do not have enough iodine [188]. Some studies in humans have found mild side effects like bloating, constipation, or stomach pain when people take more than 100 mg of isoflavones each day [204]. Therefore, while eating normal amounts of soy foods is considered safe, people should be careful when using high-dose isoflavone supplements, especially for a long time or if they have hormone-related health problems [204,205,206]. Human studies also show this. Some people had side effects like stomach pain, bloating, or constipation when they took high doses of isoflavone supplements [200,207]. For most individuals, moderate soy intake (1–2 servings/day, equivalent to 40–50 mg isoflavones) does not produce harmful effects [184]. Individuals with hormone-sensitive disorders, including endometrial, ovarian, or breast cancer, are advised to exercise caution [183,208]. Although phytoestrogens demonstrate weaker estrogen-like effects compared to natural estradiol, their ability to activate ERα remains a theoretical concern [196]. As a result, healthcare professionals commonly recommend that those with a history of hormone-related cancers avoid high-dose isoflavone supplements, while moderate consumption from regular dietary sources is generally regarded as safe [183,208].

One of the most debated issues about daidzein and genistein is their possible effect on cancer, especially hormone-sensitive cancers such as breast and prostate cancer [183,196]. Several laboratory studies (in vitro) have shown that low levels of genistein can promote the growth of estrogen receptor-positive (ER+) breast cancer cells, likely through the activation of ERα [197]. However, these findings are not always observed in preclinical or human studies [183,196]. Studies have shown that, in many Asian countries, people who consume soy from a young age tend to have a lower risk of developing breast cancer [184,209]. Wu et al. found that women who ate large amounts of soy during both adolescence and adulthood had a from 25 to 30 percent lower chance of developing breast cancer compared to those who consumed very little soy [184,209]. In contrast, studies from Western countries have shown mixed results, which may be explained by differences in soy consumption, overall diet, or genetic variations in isoflavone metabolism [184,190]. Additionally, the timing of soy exposure may play an important role. Consuming soy during early life may influence breast tissue development and help reduce the risk of developing breast cancer later in life [184,209]. In contrast, starting soy supplementation later in life may not provide the same protective effect and could have different outcomes in postmenopausal women [184,209].

For individuals with a history of hormone-sensitive breast cancer, the current consensus is to avoid high-dose isoflavone supplements [183,209]. Soy consumption is generally considered safe when included in a balanced diet and may provide health benefits, especially for people without hormone-related health issues [184]. Daidzein and genistein are among the most extensively studied phytoestrogens due to their ability to act as natural selective estrogen receptor modulators (SERMs) [24]. Their pharmacokinetic properties, including moderate bioavailability, metabolism to conjugated forms, and interindividual variation due to gut microbiota, affect their systemic effects [185,190]. These isoflavones exert tissue-selective estrogenic activities that may offer benefits, such as improved bone and cardiovascular health, especially in postmenopausal women [182,197,198]. However, because daidzein and genistein can affect hormone-sensitive organs, it is important to be cautious, especially for individuals who have or are at risk of estrogen-related cancers [183,208]. Although high-dose supplementation is not recommended for these people, recent research indicates that regularly consuming moderate amounts of soy foods is safe and may help reduce cancer risk over the long term [184,209]. Unlike HRT, which may increase the risk of hormone-related cancers, phytoestrogens could be a safer option for people with low estrogen levels. For example, a big study called the Women’s Health Initiative (WHI) found that women who took both estrogen and progestin had a 24% higher risk of breast cancer (HR 1.24; 95% CI, 1.01–1.53) [210]. Also, using estrogen alone can raise the risk of endometrial cancer by from 2 to 3 times in women who still have a uterus [211]. On the other hand, eating a lot of soy foods has been linked to a 25–30% lower risk of breast cancer in Asian women, especially when they begin eating soy at a young age and continue into adulthood [184,209]. This suggests that phytoestrogens from soy and similar foods might be safer than hormone medicine. However, more research is still needed. Long-term studies are important to know if phytoestrogens are safe, how well they work, and how they affect different kinds of people [184,190,209]. For now, daidzein and genistein can be considered safe parts of the diet when eaten in normal food amounts [184]. Overall, daidzein and genistein demonstrate favorable absorption and metabolism profiles, along with selective estrogen receptor modulation, supporting their potential health benefits with appropriate caution in hormone-sensitive conditions.

## 3. Future Aspects

While the potential of daidzein and genistein as alternatives to conventional hormone replacement therapy is evident, there are several areas that require further investigation. Pharmaceutical hormone replacement therapy (HRT) remains the most established treatment for menopausal symptoms, with proven efficacy in relieving vasomotor symptoms and preventing bone loss. However, its use is associated with an increased risk of adverse events, including breast cancer, thromboembolic disorders, and cardiovascular complications, particularly with long-term use. In contrast, natural products like soy isoflavones offer a milder estrogenic effect with a more favorable safety profile, especially when consumed at dietary levels. Nevertheless, their clinical effectiveness tends to be more variable and generally lower in potency compared to synthetic estrogens. Future studies should focus on optimizing the dosage and formulation of these phytoestrogens to enhance their bioavailability and clinical efficacy. Current research often varies in terms of the dosage and duration of treatment, highlighting the need for standardized protocols to establish effective treatment regimens. Additionally, more clinical trials with larger, diverse populations are necessary to better understand the long-term safety and efficacy of soy-derived isoflavones, particularly in hormone-sensitive conditions such as breast cancer. Personalized treatment strategies that consider genetic polymorphisms and gut microbiota composition—particularly equol production status—should be a focus of future research and clinical application to optimize therapeutic outcomes. Furthermore, exploring their molecular mechanisms through advanced pharmacogenomic approaches could help identify biomarkers that predict individual responses to treatment. Investigating synergistic effects with other natural compounds or synthetic drugs may also open new therapeutic pathways for managing menopausal symptoms and promoting overall health in postmenopausal women.

## 4. Materials and Methods

This study was based on a comprehensive literature review of daidzein and genistein as natural phytoestrogens in the context of hormone replacement therapy. Relevant information was gathered from books and peer-reviewed publications available up to May 2025. The literature search and data collection were conducted between January and May 2025 using major scientific databases, including Web of Science, Scopus, PubMed, Google Scholar, and Elsevier. Keywords used in the search strategy included “daidzein,” “genistein,” “isoflavone,” “flavonoids,” “phytoestrogen,” “natural hormone replacement therapy,” “chemical structure,” and “natural sources.”

Data were extracted from studies encompassing in silico, in vitro, in vivo, and clinical research addressing the therapeutic potential of daidzein and genistein, including their antioxidant, anti-inflammatory, neuroprotective, anti-aging, anticancer, osteoprotective, and cardioprotective effects. For the Bioactivities: Estrogenic Activity of Daidzein and Genistein section, publications published up to May 2025 were reviewed. However, for the section on Other Bioactivities of Daidzein and Genistein, the literature was limited to studies published between 2020 and 2025 to ensure the inclusion of the most up-to-date evidence available. The collected evidence was critically reviewed and systematically synthesized to provide an integrated overview.

## 5. Conclusions

Daidzein and genistein, the principal soy-derived isoflavones, have emerged as promising natural alternatives to conventional hormone replacement therapy (HRT) for the management of menopausal symptoms. Their structural similarity to E2 allows them to interact with estrogen receptors—particularly ERβ—enabling tissue-selective modulation of estrogenic activity. Extensive in vitro, in vivo, and clinical studies demonstrate their capacity to alleviate vasomotor symptoms, improve bone and cardiovascular health, and exert antioxidant and anti-inflammatory effects. However, several factors influence their clinical efficacy and safety, including dosage, bioavailability, and metabolic pathways. Importantly, individual variability in response to isoflavones must be considered. Genetic polymorphisms, such as the capacity to produce equol, a key metabolite of daidzein formed by gut microbiota, may significantly influence treatment outcomes. Additionally, diet, antibiotic use, age, and other health conditions may influence gut microbiota function and, consequently, the bioactivation of isoflavones. Personalized approaches that account for genetic differences in isoflavone metabolism, gut microbiota composition, and overall health status could help optimize the therapeutic benefits of these compounds. Despite such variability, these phytoestrogens are generally well tolerated at dietary levels. While caution is advised in hormone-sensitive populations, regular consumption of soy-based foods appears safe and may offer long-term protective effects. Their dual receptor-mediated and non-receptor-mediated mechanisms support their therapeutic potential. Continued research is essential to clarify optimal dosing, evaluate long-term safety, and develop personalized treatment strategies for menopausal women seeking plant-based therapeutic options.

## Figures and Tables

**Table 1:** Key findings in recent studies of daidzein for other bioactivities.

Biological Activities	Study Model/ Assay	Effective Dose/ Concentration	Key Findings	Reference
In silico				
Neurological	Molecular docking, Molecular dynamic simulations, and ADME properties	-	Exhibited the score of best binding pose for the complex at −5.3 Kcal/ Mol and predicted to have ability to cross the blood-brain barrier	[145]
	Molecular docking against FAAH	-	Demonstrated a binding energy of −64.77 Kcal/mol and a binding affinity of −11.77 Kcal/mol	[146]
Anti-cancer	Molecular docking targeting ERα	-	Displayed strong binding to ERα but less than genistein (docking score −8.47 Kcal/mol) and formed two hydrogen bonds with critical amino acids, specifically His-524 and Gly-521.	[147]
	Molecular docking targeting human ER	-	Exhibited a strong interaction with Human Estrogen Receptor (PDB ID: 2IOK) with binding energy of −8.82 kcal/mol and interacted with Leu346, Leu384, Leu387, Phe404, and Leu525.	[148]
In vitro				
Neurological	ThT fluorescence assay, 90° Light scattering studies, TEM, ANS fluorescence assay, tyrosine fluorescence quenching studies, and CD spectroscopy	α-Synuclein (α-syn)/Daidzein molar ratios (1:0, 1:1, 1:3, and 1:5)	Inhibited α-syn fibrillation in a concentration dependent manner via modulation of hydrophobic and hydrogen bonding interactions and delaying β-rich structure formation	[145]
	FAAH enzyme inhibitory assay	-	Inhibited FAAH activity (IC_50_ = 1.3 ± 0.13 μM)	[146]
Anti-cancer	MDA-MB-231 and MCF-7 breast cancer cell lines	-	Exhibited cytotoxicity in MDA-MB-231 (IC_50_ = 25.36 ± 0.962 μM) and MCF-7 (IC_50_ = 33.23 ± 1.043 μM). However, its effect was found associated with ferroptosis only in MDA-MB-231 cells, characterized by elevated LPO, reduced GSH/GSSG ratio, and downregulated mRNA expression of ferroptosis-regulatory genes *Gpx4* and *Fsp-1*	[149]
	MCF-7 and T47D human ERα-positive breast cancer cells	1 μM	Suppressed estrogen-induced neuroglobin expression and enhanced the pro-apoptotic effects of paclitaxel in ERα-positive breast cancer cells by activating p38 MAPK signaling	[150]
	A549 and 95D human NSCLC cells	25 μM DRIA	Inhibited proliferation and colony formation of lung cancer cells by downregulating NF-κB signaling and suppressing IL-6 and IL-8	[151]
	MIA PaCa-2 pancreatic carcinoma cells and HT-29 colon adenocarcinoma cells	200 μM	Inhibited proliferation and induced DNA damage in MIA PaCa-2 and HT-29 cancer cells in a dose-dependent manner	[152]
	SW620 colorectal cancer cells	-	Inhibited cell proliferation (IC_50_ = 23.5 ± 0.8 μM) and reduced activation of oncogenic pathways by downregulating phosphorylated ERK and AKT	[153]
Anti-osteoporotic	ATDC5 mouse chondrogenic cells	10 μM	Inhibited chondrogenic differentiation in ATDC5 cells (less potent than Genistein) and suppressed proteoglycan production and chondrogenic gene expression.	[154]
	Caco-2 intestinal epithelial cells and Saos-2 human osteoblast-like cells	0.05–1.0 mg/mL	Promoted Saos-2 cell proliferation and enhancing intracellular calcium content during osteogenic induction	[155]
Cardioprotective	Human platelets isolated from platelet-rich plasma (PRP)	12.5–50 μM	Inhibited collagen-induced human platelet aggregation by suppressing granule release (ATP, serotonin, P-selectin), TXA_2_ production, integrin αIIbβ3 activation, and key signaling pathways (PI3K/PDK1/Akt/GSK3αβ/p38, and ERK)	[156]
In vivo				
Neurological	Male and female Balb/c mice	20 mg/kg/day for 14 days, IP	Alleviated depressive-like behavior by reducing immobility time in forced swim test and lowering plasma corticosterone level	[146]
	CUMS-induced male Swiss albino mice—a model of depression	1 mg/kg/day for 21 days, PO	Reduced depressive- and anxiety-like behaviors, and improved motor coordination and memory via upregulating ERβ-dependent ERK/mTOR signaling	[157]
	Aβ42 transgenic Drosophila flies—a model of AD	1 mM in standard food for 96 h	Prolonged the lifespan of Aβ42 transgenic flies	[158]
	I/R injury in male ICR rats—a model of ischemic stroke	20 and 30 mg/kg/day for 2 weeks, IG	Improved neurological deficits, reduced infarct size and brain edema, and restored dopamine levels, mediated by inhibiting expression of cleaved caspase-3, while activating Akt/mTOR, Akt/BAD, and BDNF/CREB signaling pathways	[159]
	TBI model in male albino BALB/c mice	10 mg/kg/day for 14 days, IP	Improved neurological function, enhanced motor coordination, reduced anxiety-like behavior, alleviated mechanical allodynia, and restored blood–brain barrier integrity.	[160]
Anti-cancer	Male albino rats with DMH and DSS- induced colorectal cancer	5 and 10 mg/kg, three times/week for 8 weeks, PO	Reduced tumor progression by lowering CXCL1, AREG, MMP-9 which involved in tumor progression and metastasis, and oxidative stress markers and improved colon tissue.	[153]
	DENA and CCl_4_-induced male Wistar rats—a model of HCC	20 and 40 mg/kg/day for 8 weeks (pre-treatment), PO	Protected against HCC by improving liver function markers (ALP, ALT, AST), reducing oxidative stress and IL-6, TNF-α, CRP, lowering tumor markers (AFP, GPC3, VEGF), and restoring near-normal liver histology	[161]
Anti-osteoporotic	OVX C57BL/6 female mice—a model of postmenopausal osteoporosis	25 mg/kg, 5 days/week for 8 weeks, IG	Enhanced bone formation, inhibited osteoclast activity, and promoted H-type vessel formation via suppression of Caveolin-1 and activation of EGFR/PI3K/AKT signaling in endothelial cells	[125]
	OVX female Wistar rats—a model of postmenopausal osteoporosis	10 mg/kg/day for 6 weeks, PO	Improved bone microarchitecture, increased femoral calcium content, enhanced intestinal calcium transporter expression (TRPV5 and TRPV6 mRNA), and favorably modulated bone metabolism markers	[162]
Clinical trial				
Anti-cancer	Chinese equol-producing postmenopausal women	63 mg/day for 6 months, PO	no significant effect on bone turnover markers or inflammation compared to placebo.	[163]

Akt = Protein Kinase B; ADME = Absorption, Distribution, Metabolism, and Excretion; AFP = Alpha-fetoprotein; ANS = 8-anilino-1-naphthalenesulfonic acid; ALP = Alkaline phosphatase; ALT = Alanine transaminase; AREG = Amphiregulin; AST = Aspartate transaminase; BAD = Bcl-2-associated agonist of cell death; BDNF = Brain-Derived Neurotrophic Factor; CCl_4_ = Carbon Tetrachloride; CD = Circular dichroism; CREB = cAMP Response Element-Binding Protein; CXCL1 = CXC Motif Chemokine Ligand 1; CRP = c-reactive protein; CUMS = Chronic unpredictable mild stress; DENA = Diethylnitrosamine; DRIA = Daidzein-rich isoflavones aglycone; DSS = Dextran sodium sulfate; EGFR = Epidermal Growth Factor Receptor; ER = Estrogen Receptor; ERα = Estrogen receptor alpha, ERK = Extracellular signal-regulated kinase; FAAH = Fatty acid amide hydrolase; GPC3 = Glypican-3; GSH = Glutathione; GSK3αβ = Glycogen Synthase Kinase 3 alpha/beta; GSSG = Glutathione disulfide; HCC = Hepatocellular carcinoma; IC_50_ = Half-maximal inhibitory concentration; IG = Intragastrically; I/R = Ischemia/reperfusion; LPO = lipid peroxidation; MAPK = Mitogen-Activated Protein Kinase; mTOR = Mechanistic Target of Rapamycin; MMP-9 = Matrix Metalloproteinase 9; NSCLC = Non-small cell lung cancer; OVX = Ovariectomy or Oophorectomy; PDB = Protein Data Bank; PI3K = Phosphoinositide 3-kinase; PO = Per oral; TBI = Traumatic brain injury; TEM = Transmission electron microscopy; TXA_2_ = Thromboxane A_2_; VEGF = Vascular endothelial growth factor.

**Table 2:** Key findings in recent studies of genistein for other bioactivities.

Biological Activities	Study Model/ Assay	Effective Dose/ Concentration	Key Findings	Reference
In silico				
Neurological	Molecular docking studies against therapeutic targets for AD	-	Exhibited high binding affinities against human AChE, β-secretase, TACE, GSK3, and APP. It was also confirmed for its favorable drug-likeness profiles, although less likely to penetrate the CNS.	[164]
Anti-cancer	Molecular docking targeting ERα	-	Exhibited strong binding affinity toward ERα (−8.5 kcal/mol) and formed 5 hydrogen bonds with Leu-387, Glu-353, Arg-394, Glu-419, and His-524	[147]
	Molecular docking targeting human ER	-	Showed a favorable binding (−8.36 kcal/mol) and interacted with Leu346, Leu384, Leu387, and Phe404	[148]
In vitro				
Neurological	OGD/R-induced rat pheochromocytoma PC12 cells	30 µM	Reduced the levels of Ca^2+^, ROS, apoptosis as well as inhibited the Wnt/Ca^2+^ signaling pathway	[165]
	OGD/R-induced N9 primary microglia and the cocultured N9 with HT22 hippocampal neuronal cells	5 μg/mL	Reduced inflammatory responses (TNF-α, IL-1β, IL-18, IL-6 and cleaved caspase-1) and microglial expression of NLRP3 inflammasome	[166]
Anti-cancer	MDA-MB-231 and MCF-7 breast cancer cells	-	Exhibited cytotoxicity in MDA-MB-231 (IC_50_ = 26.72 ± 1.261 μM) and MCF-7 (IC_50_ = 45.02 ± 1.064 μM). However, its effect was found associated with ferroptosis only in MDA-MB-231 cells, characterized by elevated LPO, reduced GSH/GSSG ratio, and downregulated mRNA expression of ferroptosis-regulatory genes *Gpx4* and *Fsp-1*	[149]
	Human prostate cancer cell line DU145 and Normal prostate epithelial cells HPrEC	50–100 μM	Inhibited DU145 proliferation by inducing p53-mediated, caspase-dependent apoptosis and suppressing oncogenic STAT3, Akt, ERK, and p38 signaling pathways, with minimal cytotoxicity to normal prostate cells.	[167]
	HAG/src3-1 human gallbladder carcinoma cells (v-Src-transfected) and HAG/neo3-5 control cells	50 μM	Inhibited Src-driven gallbladder cancer cell proliferation by inducing G2/M cell cycle arrest through upregulation of p53 and p21while reducing phosphorylated p21	[168]
	Human colon cancer SW1116, DLD-1, and SW480 cell lines	75 μM	Suppressed proliferation of colon cancer cells and reactivated WNT5a expression in SW1116 cells by promoter demethylation, suggesting an epigenetic mechanism	[169]
Anti-osteoporotic	Mouse chondrogenic ATDC5 cells	10 μM	Suppressed chondrogenic differentiation in ATDC5 cells by reducing sulfated proteoglycans, collagen fibers, and calcium deposition, and downregulating genes related to chondrocyte differentiation, while promoting osteogenic marker expression	[154]
In vivo				
Neurological	I/R injury in OVX female C57BL/6 J mice—a model of postmenopausal stroke	10 mg/kg/day for 2 days, IP	Enhanced the neuronal GPER/PGC-1α pathway and inhibited NLRP3 inflammasome activation	[170]
	I/R injury in male Sprague–Dawley rats—a model of ischemic stroke	100 mg/kg/day for 21 days, PO	Alleviated CIRI by reduced infarct size, improved neurological function. It also mitigated Ca^2+^ overload, oxidative stress, and apoptosis via inhibition of the Wnt/Ca^2+^ signaling pathway.	[165]
	I/R injury in reproductively senescent female C57BL/6 J mice—a model of postmenopausal stroke	10 mg/kg/day for 2 weeks, IP	Alleviated cerebral ischemic injury by improving neurological deficit scores and reducing inflammatory responses (TNF-α, IL-1β, IL-18, IL-6, and cleaved caspase-1) as well as microglial expression of NLRP3 inflammasome	[166]
	PTZ-induced male Sprague–Dawley rats—a model of epilepsy	5 and 15 mg/kg for 30 min (pre-treatment), IP	Reduced the intensity and duration of seizures and promoted neuronal survival while inhibited microglial and astrocytic activation. The effects are mediated through the inhibition of JAK2/STAT3 signaling pathway and activation of the Keap1/Nrf2 oxidative stress pathway.	[171]
Anti-osteoporotic	Male Sprague–Dawley rats with orchiectomy-induced osteoporosis	1 g/kg in food (~20.7 mg/kg/day) for 95, 102 and 151 days, PO	Demonstrated short-term improvement in cortical bone thickness via the estrogen pathway but had limited long-term osteoprotective effects and no significant benefit on trabecular bone	[172]
	Male Sprague–Dawley rats with T2DM	30 mg/kg/day for 8 weeks, PO	Improved bone density, enhanced bone microarchitecture, promoted osteogenesis, suppressed bone resorption, and reduced inflammation in diabetic osteoporotic rats by modulating the OPG/RANKL, PPAR-γ, and β-catenin/Runx-2 pathways	[173]
	Female Wistar rats	100 mg/kg/day in combination with 10 mg daidzein/kg/day for 8 weeks, PO	Upregulated Trpv6 expression, promoting intestinal calcium transport, and decreased serum pyridinoline, a marker of bone resorption	[174]
	Female Sprague–Dawley rats with DMBA-induced mammary gland cancer	0.2 mg/kg/day for 10 weeks, PO	Disrupted bone structure, increased calcium accumulation, and altered mineral composition in rats with breast cancer, leading to fragile and structurally compromised bones.	[175]
Cardioprotective	Male Wistar rats with N^ω^-nitro-L-arginine methyl ester hydrochloride (L-NAME)-induced NO deficiency hypertension and cardiac dysfunction	80 mg/kg/day for 5 weeks, PO	Prevented NO deficiency-induced hypertension, oxidative stress, cardiac hypertrophy, and fibrosis in rats by suppressing RAS activation and the Ang II/AT_1_R/NADPH oxidase/TGF-β1 pathway	[176]
	OVX female Wistar rats—a model of menopause hypoestrogenism	15, 30 and 60 mg IGD/kg/day for 3 weeks, PO	Enhanced aortic VEGF expression, suggesting a potential cardioprotective effect through promoting vascular endothelial repair and angiogenesis	[177]
Clinical trial				
Anti-aging	Randomized, double-blind, placebo-controlled clinical trial in postmenopausal women (*n* = 50)	Product consisted of 4% genistein, TOP on facial skin twice daily for 6 weeks	Increased skin hydration, reduced fine pores and pore area, decreased wrinkles, and improved overall facial skin quality	[178]

AChE = Acetylcholinesterase; Ang II = Angiotensin II; AD = Alzheimer’s disease; Akt = Protein Kinase B; APP = Amyloid-β precursor protein; AT_1_R = Angiotensin II Type 1 Receptor, CIRI = Cerebral ischemia/reperfusion injury; CNS = Central nervous system; DMBA = 7,12-Dimethylbenz[a]anthracene; ER = Estrogen Receptor; ERK = Extracellular signal-Regulated Kinase; GPER = G protein-coupled estrogen receptor; GSH = Glutathione; GSK3 = Glycogen synthase kinase 3; GSSG = Glutathione Disulfide; I/R = Ischemia/reperfusion; IC_50_ = Half-maximal inhibitory concentration; IGD = Isoflavone Genistein and Daidzein combination; IL = Interleukin; IP = Intraperitoneally; NLRP3 = Nod-like receptor protein 3; NO = Nitric oxide; OGD/R = Oxygen-glucose deprivation/reoxygenation; OPG = Osteoprotegerin; OVX = Ovariectomy or Oophorectomy; PGC-1α = Peroxisome proliferator-activated receptor-gamma coactivator 1α; PPAR-γ = Peroxisome proliferator-activated receptor-γ, PTZ = Pentylenetetrazole; RANKL = Receptor activator of nuclear factor κB ligand; RAS = Renin–Angiotensin System; STAT3 = Signal Transducer and Activator of Transcription 3; Runx-2 = Runt-related transcription factor 2; T2DM = Type 2 diabetes mellitus; TACE = Tumor necrosis factor-α converting enzyme; TGF-β1 = Transforming Growth Factor Beta 1; Trpv6 = Transient Receptor Potential Vanilloid 6; TOP = Topical administration; VEGF = Vascular Endothelial Growth Factor.

## Data Availability

Not applicable.

## Supplementary Materials

The following supporting information can be downloaded at: https://www.mdpi.com/article/10.3390/ijms26146973/s1, References [157,158,160,172,212,213,214] are cited in the supplementary materials.

## Abbreviations

The following abbreviations are used in this manuscript:

17β-HSD	17β-Hydroxysteroid Dehydrogenase
AChE	Acetylcholinesterase
AD	Alzheimer’s disease
ADME	Absorption, Distribution, Metabolism, and Excretion
AF-1/AF-2	Activation Function-1/Activation Function-2
AFP	Alpha-fetoprotein
Akt	Protein Kinase B
ALP	Alkaline phosphatase
ALT	Alanine transaminase
AMPK	AMP-Activated Protein Kinase
Ang II	Angiotensin II
ANS	8-anilino-1-naphthalenesulfonic acid
APP	Amyloid-β precursor protein
AREG	Amphiregulin
AST	Aspartate transaminase
AT_1_R	Angiotensin II Type 1 Receptor
BAD	Bcl-2-associated agonist of cell death
BDNF	Brain-Derived Neurotrophic Factor
CAT	Catalase
CCl_4_	Carbon Tetrachloride
CD	Circular dichroism
CIRI	Cerebral ischemia/reperfusion injury
CNS	Central nervous system
CREB	cAMP Response Element-Binding Protein
CRP	c-reactive protein
CUMS	Chronic unpredictable mild stress
CXCL-12	C-X-C Motif Chemokine Ligand 12
CXCL1	CXC Motif Chemokine Ligand 1
CYP3A4	Cytochrome P450 3A4
DBD	DNA-Binding Domain
DENA	Diethylnitrosamine
DMBA	7,12-Dimethylbenz[a]anthracene
DMS	Dimethylhydrazine dihydrochloride
DRIA	Daidzein-rich isoflavones aglycone
DSS	Dextran sodium sulfate
EGFR	Epidermal Growth Factor Receptor
EGR-1	Early Growth Response Protein 1
ER	Estrogen Receptor
ERK	Extracellular signal-regulated kinase
ERα	Estrogen Receptor Alpha
ERβ	Estrogen Receptor Beta
FAAH	Fatty acid amide hydrolase
FoxM1	Forkhead Box M1
GLUT1/GLUT4	Glucose Transporter 1/4
GnRH	Gonadotropin-Releasing Hormone
GPC3	Glypican-3
GPER	G-protein-coupled Estrogen Receptor
GPER	G protein-coupled estrogen receptor
GSH	Glutathione
GSK3	Glycogen synthase kinase 3
GSK3αβ	Glycogen Synthase Kinase 3 alpha/beta
GSSG	Glutathione disulfide
GST	Glutathione S-transferases
H_2_O_2_	Hydrogen Peroxide
HCC	Hepatocellular carcinoma
HMGB1	High mobility group box 1
HRT	Hormone Replacement Therapy
I/R	Ischemia/reperfusion
IC_50_	Half-maximal inhibitory concentration
IG	Intragastrically
IGD	Isoflavone Genistein and Daidzein combination
IL	Interleukin
IP	Intraperitoneally
LBD	Ligand-Binding Domain
LFPI	Lateral fluid percussion injury
LPO	lipid peroxidation
MAPK	Mitogen-Activated Protein Kinase
MAPK/ERK	Mitogen-Activated Protein Kinase/Extracellular Signal-Regulated Kinase
MCAO	Middle cerebral artery occlusion
MKRN3	Makorin Ring Finger Protein 3
MMP-9	Matrix Metalloproteinase 9
MnSOD	Manganese Superoxide Dismutase
MPO	Myeloperoxidase
mTOR	Mechanistic Target of Rapamycin
NLRP3	Nod-like receptor protein 3
NO	Nitric oxide
NSCLC	Non-small cell lung cancer
OGD/R	Oxygen-glucose deprivation/reoxygenation
OPG	Osteoprotegerin
OVX	Ovariectomy or Oophorectomy
PDB	Protein Data Bank
PGC-1α	Peroxisome Proliferator-Activated Receptor Gamma Coactivator 1-Alpha
PGC-1α	Peroxisome proliferator-activated receptor-gamma coactivator 1α
PGE2	Prostaglandin E2
PI3K	Phosphatidylinositol 3-Kinase
PKCγ	Protein Kinase C Gamma
PO	Per oral
PPAR-γ	Peroxisome proliferator-activated receptor-γ
PTZ	Pentylenetetrazole
RANKL	Receptor activator of nuclear factor κB ligand
RAS	Renin–Angiotensin System
RCT	Randomized Controlled Trial
ROS	Reactive Oxygen Species
Runx-2	Runt-related transcription factor 2
SC	Subcutaneously
SERM	Selective Estrogen Receptor Modulator
SIRT1	Sirtuin 1
SOD	Superoxide dismutase
SRC3	Steroid Receptor Coactivator 3
STAT3	Signal Transducer and Activator of Transcription 3
SULTs	Sulfotransferases
T2DM	Type 2 diabetes mellitus
TACE	Tumor necrosis factor-α converting enzyme
TBARS	Thiobarbituric Acid Reactive Substances
TBI	Traumatic brain injury
TEM	Transmission electron microscopy
TGF-β1	Transforming Growth Factor Beta 1
TOP	Topical administration
Trpv6	Transient Receptor Potential Vanilloid 6
TXA_2_	Thromboxane A_2_
UGTs	UDP-Glucuronosyltransferases
VEGF	Vascular endothelial growth factor
VMS	Vasomotor Symptoms
YES assay	Yeast Estrogen Screen Assay
