# Supplementary material for: Daidzein and Genistein: Natural Phytoestrogens with Potential Applications in Hormone Replacement Therapy
Source: Int J Mol Sci. 2025 Jul 20;26(14):6973. doi: 10.3390/ijms26146973 (PMC12294992; doi:10.3390/ijms26146973)
Supplement: Supplementary file 1 [file ijms-26-06973-s001.zip › ijms-3693557-supplementary.pdf]

Table S1 Key Findings in Recent Studies on Antioxidant and Anti-inflammatory activities of Diadzein and Genistein.

Biological Activities	Compounds	Study Model/ Assay	Effective Dose/ Concentration	Key Findings	Reference
In silico					
Anti-inflammatory	Genistein	Molecular docking and molecular dynamics simulations combined with multi-spectroscopic approaches against HMGB1	-	Showed direct interaction with HMGB1, a key protein involved in mediating neuroinflammatory responses	[214]
In vitro					
Antioxidant	Diadzein	H_2O_2-induced HT-22 mouse hippocampal neuronal cells	1–10 μM	Reduced NO generation and enhanced cell viability	[160]
	Genistein	H_2O_2, DPPH•, and conjugated diene scavenging activity and TBARS assay	-	Exhibited scavenging activities against H_2O_2 (IC_{50} = 56.9 ± 0.12 μg/mL), DPPH• (IC_{50} = 50.25 ± 1.22 μg/mL), and conjugated diene (IC_{50} = 38.05 ± 0.44 μg/mL), and TBARS inhibition (IC_{50} = 65.1 ± 0.4 μg/mL)	[24]
Anti-inflammatory	Diadzein	LPS-stimulated murine peritoneal macrophages	100 μM	Inhibited inflammatory responses by downregulating NO and PGE2 generation, as well as inhibiting NLRP3, IL-1β, IL-18 expressions in the inflammasome pathway	[215]
	Diadzein	Palmitic acid-induced C2C12 myotube cells	25 μM	Reduced inflammatory cytokines (TNF-α and IL-6), and suppressed the expression of muscle atrophy-related genes and proteins (Fbxo32, Trim63, Hdac4, Foxo1, and MuRF1)	[215]

	Genistein	HMGB1-treated BV2 microglia cells	50 μM	Mitigated neuroinflammation by suppressing about 42% of NO release	[216]
In vivo					
Antioxidant	Diadzein	CUMS-induced male Swiss albino mice-a model of depression	1 mg/kg/day for 21 days, PO	Increased the levels of SOD, CAT, and AChE in the cortex, hippocampus, and medulla of the CUMS mouse model	[157]
	Diadzein	H_2O_2-induced female Drosophila flies	1 mM in standard food for 96 h	Promoted survival under oxidative damage, reduced ROS increased SOD, CAT, GSH levels, and restored decreased expression of several antioxidant genes	[158]
	Diadzein	TBI model in male albino BALB/c mice	10 mg/kg/day for 14 days, IP	Increased antioxidant enzyme levels (SOD, CAT, GSH, and GST), and decreased the levels of oxidative stress markers (NO, MPO, and LPO)	[160]

CAT = Catalase; CUMS = Chronic unpredictable mild stress; GST = Glutathione S-transferases; H_2O_2 = Hydrogen peroxide; LPO = lipid peroxidation; MPO = Myeloperoxidase; NO = Nitric oxide; PGE2 = Prostaglandin E2; SOD = Superoxide dismutase
